# Recent advances in modeling turbulent wind flow at pedestrian-level in the built environment

**DOI:** 10.1007/s44223-022-00008-7

**Published:** 2022-07-18

**Authors:** Jiading Zhong, Jianlin Liu, Yongling Zhao, Jianlei Niu, Jan Carmeliet

**Affiliations:** 1 College of Environmental Science and Engineering, Donghua University, Shanghai, China; 2 Chair of Building Physics, Department of Mechanical and Process Engineering, ETH Zürich, 8092 Zürich, Switzerland; 3 Department of Building Environment and Energy Engineering, The Hong Kong Polytechnic University, Kowloon, Hong Kong SAR China

**Keywords:** Pedestrian-level wind (PLW), Computational fluid dynamics (CFD), Data-driven model, Steady- and unsteady-state simulations, Uncertainty quantification

## Abstract

Pressing problems in urban ventilation and thermal comfort affecting pedestrians related to current urban development and densification are increasingly dealt with from the perspective of climate change adaptation strategies. In recent research efforts, the prime objective is to accurately assess pedestrian-level wind (PLW) environments by using different simulation approaches that have reasonable computational time. This review aims to provide insights into the most recent PLW studies that use both established and data-driven simulation approaches during the last 5 years, covering 215 articles using computational fluid dynamics (CFD) and typical data-driven models. We observe that steady-state Reynolds-averaged Navier-Stokes (SRANS) simulations are still the most dominantly used approach. Due to the model uncertainty embedded in the SRANS approach, a sensitivity test is recommended as a remedial measure for using SRANS. Another noted thriving trend is conducting unsteady-state simulations using high-efficiency methods. Specifically, both the massively parallelized large-eddy simulation (LES) and hybrid LES-RANS offer high computational efficiency and accuracy. While data-driven models are in general believed to be more computationally efficient in predicting PLW dynamics, they in fact still call for substantial computational resources and efforts if the time for development, training and validation of a data-driven model is taken into account. The synthesized understanding of these modeling approaches is expected to facilitate the choosing of proper simulation approaches for PLW environment studies, to ultimately serving urban planning and building designs with respect to pedestrian comfort and urban ventilation assessment.

## Introduction

Building sustainable and healthy cities has become an important attention point on the international agenda being continuously underscored in future. As stated in a United Nations report, 55% of the global population are currently living in cities. The percentage is projected to increase to 68% by the year 2050 (UN, 2018). Meanwhile, current urban developments and densifications have led to various urban health problems, e.g. increased lung cancer risk due to excessive vehicle waste exposure (Scungio et al., 2018), increased influenza infection risk due to lateral and upwind spread of virus (Wei et al., 2018), and deteriorated outdoor thermal comfort due to high pedestrian-level wind (PLW) velocities during cold (Shui et al., 2018) or low PLWs during hot weather, especially heatwaves (Jay et al., 2021; Kubilay et al., 2020; Mei & Yuan, 2022; Moonen et al., 2012). Previous studies have proved that these aforementioned problems can to some extent be mitigated through appropriate urban planning and designs. In particular, building elevated design (Liu et al., 2017; Liu, Zhang, et al., 2019), building arcade design (Wen et al., 2017), building overhang design (Hang, Chen, et al., 2018), pocket park design (Zhong et al., 2022), and city-scale ventilation corridors (Wang et al., 2020) can make partial improvements to the local or even the urban PLW conditions.

Since the PLW flow field is located at the lower level of the urban canopy layer (UCL), it is largely affected by the geometrical features of the surrounding built environment (Blocken et al., 2004; Blocken et al., 2008; Blocken et al., 2012; Blocken & Carmeliet, 2008). Note that winds at the pedestrian level vary from one location to another, requiring high-resolution investigation approaches. Field measurements and wind tunnel tests have been conducted in several studies providing valuable data for PLW assessments (Allegrini, 2018; Moonen et al., 2007; Tominaga & Shirzadi, 2021; Zhao et al., 2021; Zou et al., 2021), but the former usually provide discrete data points and the latter are time and resource expensive. As noted in the review on computational wind engineering (Blocken, 2014), a distinct feature of numerical simulations is that they can provide whole-flow field data at practical costs, i.e. data on the relevant parameters in all points of the computational domain, suitable for detailed investigations as well as parametric design studies.

However, unvalidated simulations may be inaccurate and lead to non-optimal urban planning and design decisions, which in turn could undermine PLW studies’ credibility. Toparlar et al. (2017) documented that 58% of their surveyed urban microclimate studies by computational fluid dynamics (CFD) did not validate their simulation results against experimental data. Sometimes, even if the simulations are validated, they may in effect still be problematic. For instance, studies using reduced-scale experimental data to validate their simulations for full-scale cases could be insufficiently correct in the case of narrow street canyons due to nonlinear growth of thermal layers on building surfaces (Zhao et al., 2020). Inappropriate validations due to the absence of full-scale measurement data may lead to an imperfect interpretation of simulation results manifesting the epistemic uncertainty of simulations introduced by a lack of knowledge. Even after choosing validation cases carefully, there still are other epistemic uncertainties, for instance, the lack of knowledge for proper turbulence modeling. Fortunately, the epistemic uncertainty associated with turbulence modeling can be analyzed and minimized through verification of the models using parametric and non-parametric methods (Xiao & Cinnella, 2019). The former method focuses on comparisons between different model coefficients (e.g. turbulence model’s closure coefficient sensitivity test) and between different models (e.g. multi-model comparison studies concerning different turbulence models). The latter method focuses on critically evaluating the assumptions made by the models (e.g. eddy-viscosity approximation for the Reynolds stress). Since the parametric method has gained much popularity among different PLW simulation studies, this review will focus on how parametric methods can help to improve the PLW simulation studies’ reliability.

Recently, a variety of simulation approaches in PLW studies emerged improving both speed and accuracy. Examples are the hybrid LES-RANS (large eddy simulation & Reynolds-averaged Navier-Stokes) approach (Liu & Niu, 2016, 2019), the massively parallelized (mass. Para.) LES approach (Wang et al., 2021), and the lattice-Boltzmann method (LBM) approach (Jacob & Sagaut, 2018) used to investigate urban airflows at the pedestrian level. Apart from the established CFD models, successful applications of highly efficient data-driven models, along with the emerging trend of machine learning, appeared in PLW studies (Xiang, Zhou, et al., 2021). It is undeniable that this thriving trend of developing and applying high-efficiency simulation approaches has huge potential in promoting PLW studies, especially in terms of improving realistic urban planning and design. These simulation approaches have also widened and enriched the speed-accuracy spectrum for different PLW simulation approaches. It is meaningful to review the recent advances in this spectrum and highlight some approaches at certain locations in this spectrum.

To this end, we review PLW studies published in recent 5 years that used simulation approaches. A systematic search was conducted in the Web of Science core collection database to obtain the studies within this scope. The present review is organized as follows: 1) an introduction to the recent progress in PLW simulation studies; 2) an overview of PLW simulation studies published in recent 5 years; 3) PLW simulation studies that used steady-state simulation approaches; 4) PLW simulation studies that used unsteady-state simulation approaches; 5) a thematic discussion focusing on the applicability of unsteady-state simulation approaches in PLW comfort assessment; and 6) conclusions and limitations.

## PLW simulation studies published in the recent 5 years

In total, we have investigated 215 journal articles published in recent 5 years (2017 ~ 2021). All of the articles apply CFD to study urban wind flows at the pedestrian level. These characteristics are used in query search in the Web of Science core collection database. It is possible that there are other studies that have also made essential contributions to the PLW simulation approaches but are not covered in this review. These could include conference papers, studies that are not indexed in the Web of Science core collection database, studies that do not emphasize their pedestrian-level applications, and studies that are published later than the publication year included in this review. It is important to note that publications published earlier than 5 years ago are not included in this review as we attempt to discuss the most recent advances in simulation approaches to underpin sound and efficient decision-makings for PLW simulation studies. However, we refer to some of these older publications when needed.

**Fig. 1:**
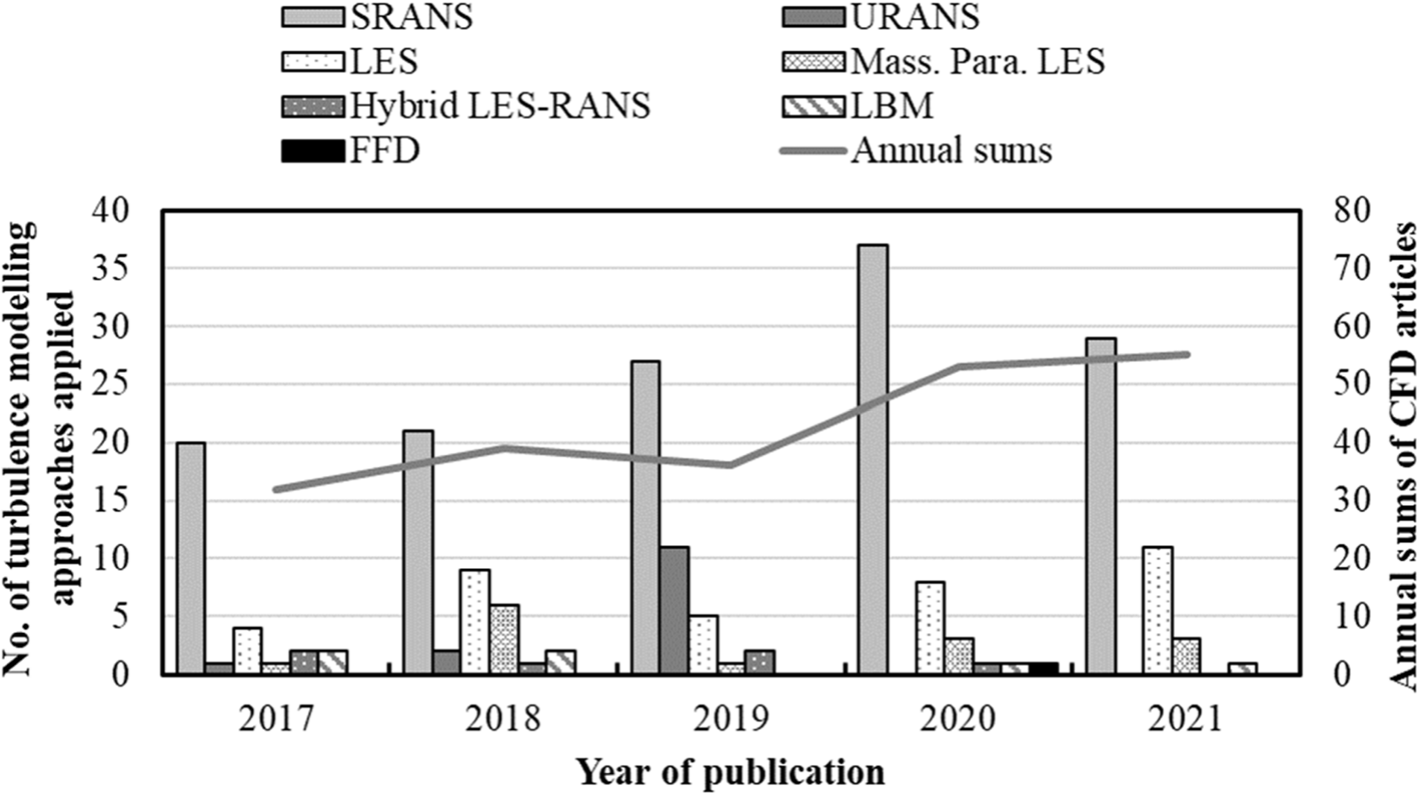
CFD applications in PLW studies published in recent 5 years

The reviewed journal articles’ distribution is plotted in terms of their publication year and their specific turbulence modeling approach, as shown in Fig. 1. Besides, we also document some typical data-driven approaches, e.g. neural networks and non-intrusive reduced-order modeling (NIROM) models, as they represent emerging trends in current PLW studies. Data-driven articles are presented and discussed in sections 3.2 and 4.2.

Figure 1 shows that the applications of CFD in PLW studies are gradually increasing within recent 5 years. Specifically, the turbulence modeling approaches are steady-state RANS (SRANS, e.g. in Tsichritzis and Nikolopoulou (2019) and Shirzadi et al. (2017)), unsteady-state RANS (URANS, e.g. in Antoniou et al. (2019) and Sanchez et al. (2021)), LES (e.g. in Zhang, Kwok, et al. (2021) and Liu, Zhang, et al. (2019)), mass. para. LES (e.g. in Wang et al. (2021) and Zhang, Ye, et al. (2021)), hybrid LES-RANS (e.g. in Liu et al. (2017) and Vita et al. (2020)), LBM (e.g. in Ahmad et al. (2017) and Han et al. (2021)), and fast fluid dynamics (FFD, in Mortezazadeh and Wang (2020)). The most popular modeling approach in the investigated articles is still SRANS. There are 134 studies using SRANS which account for 62.3% of all the articles we investigate. Another important observation is that LES is applied in 37 studies, accounting for 17.2% of the articles. And together with the other transient modeling approaches, they constitute 37.7% of the articles. This observation differs from an earlier review by Toparlar et al. (2017), who investigated 183 CFD microclimate studies published between 1998 and 2015 and reported that 96% of the studies used SRANS approach. The less predominant use of SRANS as observed in this review probably indicates advances in computational capacities, increased availability of high-efficiency simulation approaches, and more awareness of the significance of using transient simulations.

## Steady-state simulation approaches

### Turbulence modeling

The popular use of SRANS in PLW studies partly results from the norms and best practice guidelines established in earlier studies. For example, several works in the 2000s have discussed uncertainty issues such as horizontal homogeneity (Blocken, Carmeliet, & Stathopoulos, 2007; Blocken, Stathopoulos, & Carmeliet, 2007) and model sub-configuration validation (Blocken & Carmeliet, 2008), which then led to the formulation of more general model development frameworks (Blocken, 2015; Blocken & Gualtieri, 2012). Well-recognized guidelines, namely the Cooperation in Science and Technology (COST) Action 732 (Franke et al., 2007) and the Architectural Institute of Japan’s (AIJ) best practice guideline (Tominaga et al., 2008) have been fueling the popularity of proper application of SRANS in PLW studies. Among the studies investigated in this review, SRANS is broadly applied to investigate PLW flow fields in different urban settings, ranging from an isolated building (Huang et al., 2021; Jia et al., 2021; van Druenen et al., 2019; Weerasuriya et al., 2018; Zhang, Yang, et al., 2021) to more complex geometries such as building arrays (Allegrini & Carmeliet, 2017; Hang, Chen, et al., 2018; Hang, Xian, et al., 2018; Lin et al., 2019; Sattar et al., 2018; Sha et al., 2018), generic street canyons (Liu et al., 2021; Sun & Zhang, 2018; Wen & Malki-Epshtein, 2018; Yang et al., 2020; Zhang et al., 2019; Zhang, Chen, et al., 2020), and realistic urban areas (Ricci et al., 2020; Santiago et al., 2021; Sousa & Gorle, 2019; Tsichritzis & Nikolopoulou, 2019; Vervoort et al., 2019).

The SRANS equation is an approximate form of the Navier-Stokes equation (NSE), which uses the Reynolds averaging process to average out the fluctuation velocities. Specifically, this process introduces a stress term known as the Reynolds stress. The Reynolds stress is a tensor of fluctuation velocities which are not solved for in SRANS simulations. In order to make the Reynolds averaged NSE solvable, turbulence models are required to close the set of equations. The present review identified the top three most commonly used turbulence models in the SRANS-based PLW studies. They are the standard (STD) *k*-*ε*, the re-normalization group (RNG) *k*-*ε*, and the realizable (RLZ) *k*-*ε* turbulence models, which account for 43%, 23%, and 17% of the turbulence models used in SRANS studies investigated in this review, respectively.

**Fig. 2:**
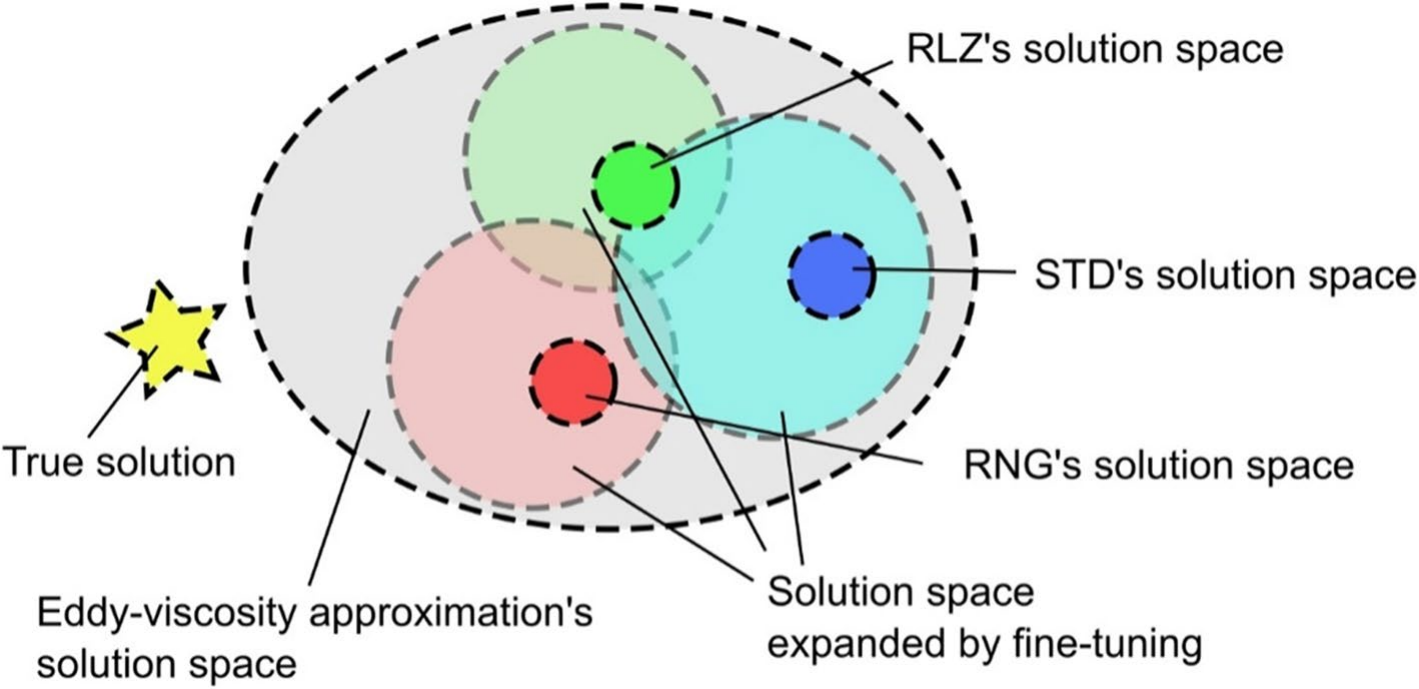
A conceptual illustration of the solution space reachable by the *k-ε* turbulence models and potential accuracy improvements from fine-tuning and multi-model methods (inspired by Xiao and Cinnella (2019))

The popular use of the STD *k*-*ε* model does not necessarily mean that the model is more accurate. Typical values for the closure coefficients involved in the model are determined from an earlier study by Launder and Spalding (1974), who focused on canonical flow problems such as free shear flow and channel flow. However, it has been well documented in earlier studies that the STD *k*-*ε* model underpredicts turbulence kinetic energy (TKE) in both the separation region over the roof and the wake region behind an isolated building, resulting in inaccurate prediction of the isolated building’s wake region length (Gousseau et al., 2011; Mochida & Lun, 2008; Vardoulakis et al., 2011; Yoshie et al., 2007). By fine-tuning the closure coefficients of *C*_ε1_, *C*_ε2_, *σ*_ε_, *σ*_k_, and mostly *C*_μ_, it is possible to increase the production of TKE and shorten the predicted wake length, hence improving prediction accuracy (Shirzadi et al., 2017). Nevertheless, achieving desired fine-tuning outcomes requires expertise in modeling turbulence flow to some extent. Besides, the intrinsic model structure would limit the solution space reachable by fine-tuning of the coefficients. As shown in Fig. 2, fine-tuning will unlikely improve the prediction accuracy because the true solution might locate outside the solution space (Xiao & Cinnella, 2019). More accurate predictions may be achieved when switching to the *k*-*ε* model variants, which incorporate different considerations regarding the production of TKE and its dissipation rate. The RNG *k*-*ε* model incorporates contributions of the mean flow field’s strain-rates to the production of TKE, and thus has more realistic predictions of TKE in separation regions. Good agreement with wind tunnel test results is obtained in several studies that focus on the airflow around isolated buildings using the RNG *k*-*ε* turbulence model (Bairagi & Dalui, 2021; Lee & Mak, 2022; Li & Chen, 2020; Liu, Wu, et al., 2020). Several other studies applied the RLZ *k*-*ε* turbulence model to simulate the airflows around isolated buildings (Chen & Mak, 2021; van Druenen et al., 2019; Weerasuriya et al., 2020; Zhang, Weerasuriya, et al., 2020). However, the predictions are still not fully accurate, as shown in Fig. 2, due to the underlying uncertainty from the eddy-viscosity approximation.

After all, fine-tuning and multi-model methods induce extra computational cost, which to some extent undermines the cost-effectiveness of SRANS. However, these extra computational costs are inevitable due to the lack of predictive generality of SRANS. The lack of predictive generality means that, for instance, a given turbulence model is only valid for a limited number of validated problems. As an example, the RNG *k*-*ε* model obtains a better accuracy for modeling turbulent airflows around an isolated building, compared to RLZ *k*-*ε* model with a Reynolds number (*Re*) of 3.7 × 10^4^ (Lee & Mak, 2022), but inferior accuracy with a *Re* of 4.2 × 10^4^ (van Druenen et al., 2019).

### Data-driven models

**Fig. 3:**
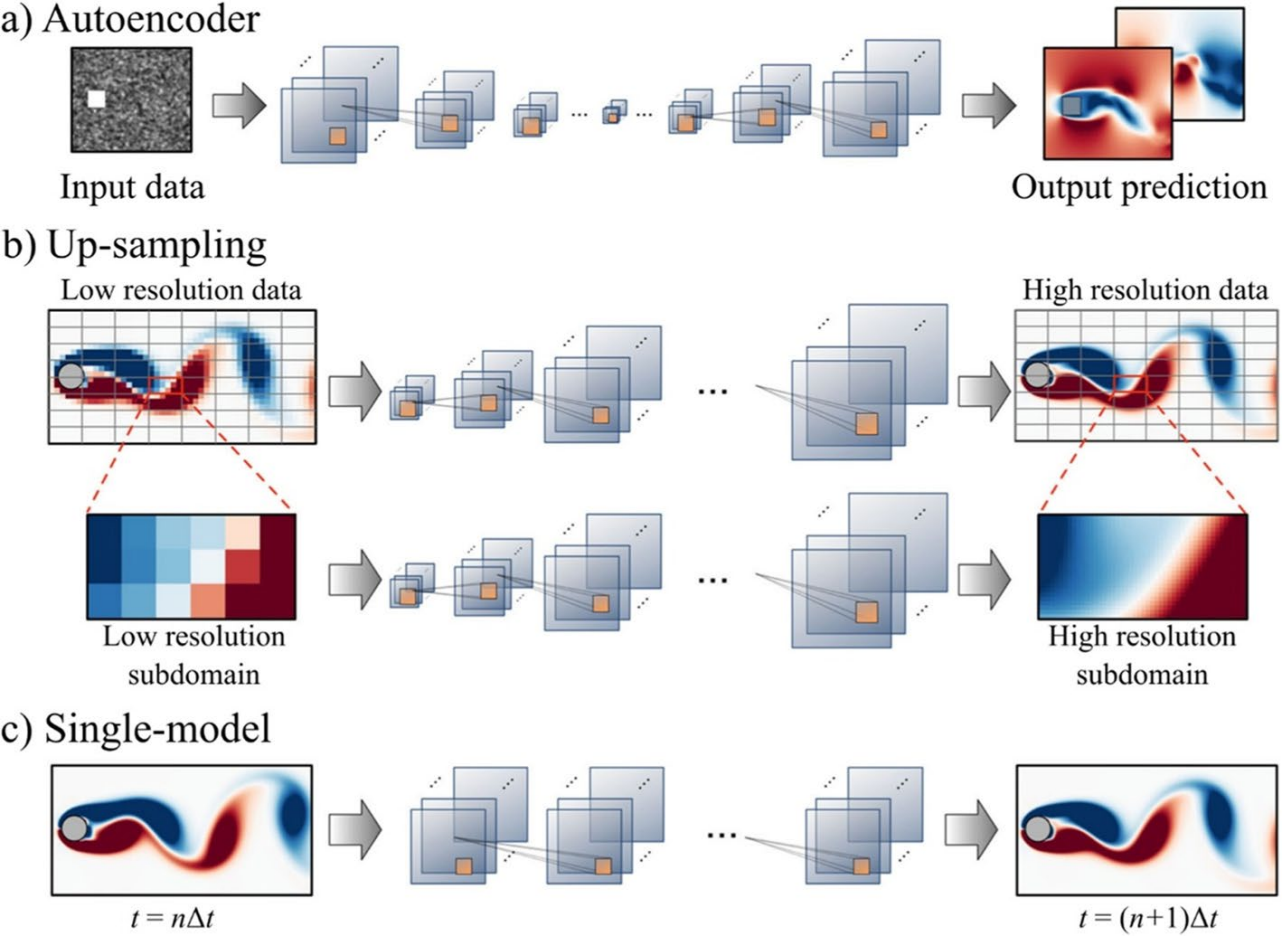
Model architectures for potential applications of generative data-driven models in PLW studies (modified from Morimoto et al. (2022))

Applying machine learning in fluid mechanics has become an active yet still challenging topic (Brunton et al., 2020). Among the variety of models, generative models have drawn our attention. They are capable of predicating PLW flow fields in an extremely efficient way having a great potential for making a difference to both academic and practical built environment applications. There are different generative model architectures that can be used for PLW studies, as shown in Fig. 3. We will mainly cover the autoencoder architecture as it is believed to have versatile performance and thus promising applicability. More information on up-sampling and single-model architectures can be found in Calzolari and Liu (2021) and Fukami et al. (2019).

The autoencoder model architecture is also often referred to as non-intrusive reduced order model or NIROM, which will be introduced in section 4.2. In this section, the main characteristics of the autoencoder architecture are discussed. Typically, an autoencoder contains an encoder and a decoder. The encoder downsizes the high-dimensional inputs into a low-dimensional latent vector, which can be effectively interpreted by the decoder. Then, the decoder upscales the latent vector to predict new data with the same dimensions of the input data. The examples given in Fig. 3 are based on CNNs (convolutional neural networks). In general, the CNN model uses a convolution kernel to slide through each input data (usually pixels) to get outputs, which serve as inputs for the next layer. The convolution kernel contains all weights needed for the current inputs, which is an efficient data storage method. In PLW studies where the flow fields are often presented and analyzed in the form of image-like contour plots, CNN models have been applied for flow field prediction purposes (Mokhtar et al., 2020; Xiang, Fu, et al., 2021; Xiang, Zhou, et al., 2021).

**Fig. 4:**
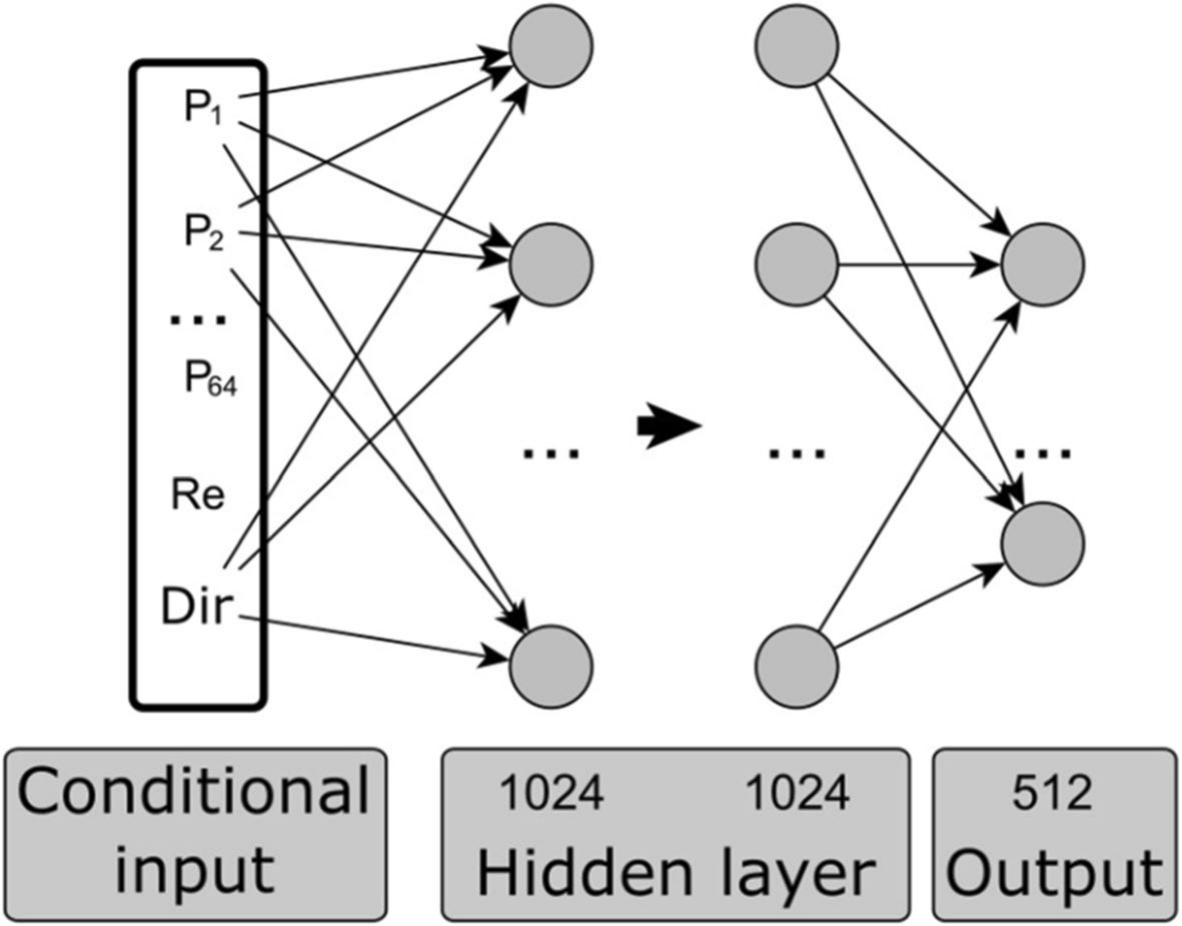
Schematic representation of a MLP model. P_1_ ~ P_64_ are geometrical information, Re is the Reynolds number, and Dir is the inflow direction (modified from Chen et al. (2020))

Multilayer perceptron (MLP) is also a popular approach to construct machine learning models. However, since each node in the MLP needs to store weights for nodes of the previous layer, dealing with high-dimensional inputs like a PLW flow field using MLPs will imply gigantic weight matrices, adding to computational and storage costs. In view of the obvious disadvantage, MLP’s application in PLW and other fluid flow studies is limited. For instance, MLP is used to extract features from boundary conditions and other flow parameters, as shown in Fig. 4. Then, the MLP’s output is fed to a generator of flow fields (Chen et al., 2020).

A common challenge for the data-driven models is the difficulty in obtaining adequately large high-fidelity training data sets. Especially, different training data sets have to be created when different urban morphologies are used, which can become computationally very expensive. Apart from tending to high-efficiency simulation approaches, this review observes a trend to apply methods that can facilitate the process of data training. There are methods that can be used to bulk out the high-fidelity training data sets. They are the flip method, the noise addition method, and the local transfer method. Nevertheless, these methods have their limits, and more details of these training data bulking methods can be found in Morimoto et al. (2022).

Another challenge for training the data-driven models is constructing appropriate loss functions. In the case of generative models for images, manually constructing loss functions is a cumbersome and challenging task (Isola et al., 2017). In the influential work done by Goodfellow et al. (2014), the generative adversarial network (GAN) is proposed. GAN avoids the manual construction of loss functions, and instead, puts two models in an adversarial game. Based on the GAN architecture, several variants are proposed that consider conditional inputs (cGAN) (Chen et al., 2020; Isola et al., 2017), which can be used to inform the data-driven models of the building geometries so PLW flow fields can also be predicted with GANs (Kim et al., 2021; Mokhtar et al., 2020). Moreover, the loss function can be used to inform the data-driven models of existing physical laws to be conserved, such as conservation of mass and momentum, so their predictions are physically realistic and accurate. Examples for this method are the physics-informed deep neural network (FlowDNN) by Chen et al. (2022) and the physics-informed neural network (PINN) by Raissi et al. (2019).

## Unsteady-state simulation approaches

### Turbulence modeling

#### Unsteady-state Reynolds-averaged Navier-stokes

The URANS approach is adapted from the SRANS approach, showing similar computational efficiency and uncertainties as introduced by turbulence models. Solving the URANS equations involves the solving of time-varying mean flows. Intermediate time scales need to be selected carefully for URANS simulations, so that the time-varying mean flows can be observed and the turbulent fluctuations are averaged out. This review investigates several studies that have conducted URANS simulations concerning PLW flow fields in various urban settings. Antoniou et al. (2019) modeled a compact urban area (0.247 km^2^) within the city Nicosia, Cyprus. Their URANS simulation included 1.5 × 10^7^ cells and modeled the time interval of 5 days with a time step size of 1 hour. Results indicate in general good agreement with the hourly measurement data. However, since the simulation adopted a large time step, mean flow variations within the one-hour time frame induced by synoptic trends are not captured. In another study (Sanchez et al., 2021), a much shorter time interval and time step size were used, namely 1 hour and 1 second, which cannot average out turbulent fluctuations and might lead to contaminated mean flows. However, the authors did not perform a systematic validation for the PLW velocities. Since shortening the time interval and the time step size can enable URANS simulations to capture more details about how the mean flows vary with time at the pedestrian level, testing the sensitivity towards different time scales for the URANS approach remains a meaningful yet insufficiently investigated task.

#### Scale resolving simulations

##### Large-eddy simulation

LES is a well-recognized high-fidelity CFD simulation approach. It is also well-known that the LES approach usually requires a large amount of computational resources. Thanks to the continuous advances in computational power, this review shows that 17.2% of the studies have applied the LES approach to the studies of the PLW flow fields in different urban settings in the recent five years. There are studies focusing on simplified geometries of isolated buildings (Liu, Yu, et al., 2020; Tse et al., 2020; Zhang, Ooka, & Kikumoto, 2020), generic urban settings of building arrays (Freidooni et al., 2021; Ikegaya et al., 2017; Ishida et al., 2018; Liu, Niu, et al., 2019; Liu, Zhang, et al., 2019), street canyons (Duan et al., 2020; Puigferrat et al., 2021; Salim et al., 2020), and complex urban areas (Adamek et al., 2017; Antoniou et al., 2017; Zhang, Kwok, et al., 2021). In some earlier studies, researchers considered different aspects of reality in their simulations. The realistic elements are also observed in the studies investigated. They include roof types (Liu, Yu, et al., 2020), parked cars (Gallagher & Lago, 2019), elevated walkways (Duan et al., 2020), tree crowns (Matsuda et al., 2018), building balconies (Zheng et al., 2022), and terraced houses (Salim et al., 2020). These realistic elements either connect to design features or human activities that are common in the current urban environment. As reported by (Zheng et al., 2020; Zheng et al., 2021), some of the realistic elements cost high simulating time of LES to achieve adequate simulation accuracy. Nevertheless, the simulation cost is justifiable given that incorporating them in simulations not only helps close the gap between simulation results and real-world processes, but also helps make the simulation results easier for urban planners and designers to interpret.

Even though the LES approach is less sensitive to uncertainties introduced by turbulence models than the RANS approach, it is prone to uncertainties originating from initial and boundary conditions or introduced by discretization schemes. This review investigates several studies that contributed to the quantification of the uncertainties in simulating PLW flow fields using the LES approach. Firstly, since the LES approach resolves most of the turbulent fluctuations, the effect of subgrid-scale (SGS) models is assessed to be minor. As reported in some earlier studies, the SGS model coefficients are less influential factors in flow field simulations. For example, the coefficient *C*_S_, when its standard value of 0.1 is used and when it is dynamically determined, is reported to have similar prediction accuracies (Ai & Mak, 2015; Gousseau et al., 2013). Recently, the study by Liu, Niu, et al. (2019) investigated the sensitivity of LES to four different SGS models simulating the PLW flow field around a generic building array. The SGS models are the standard Smagorinsky-Lilly model (SSL), the dynamic Smagorinsky-Lilly model (DSL), the wall-adapting local eddy-viscosity model (WALE), and the dynamic kinetic energy model (DKE). They reported marginal differences between the correlation coefficient (*R*) values achieved for mean velocities using different SGS models. Even though notable deviations between the different simulation results for second-order turbulence statistics are observed, they did not evaluate the results in terms of accuracy due to the lack of corresponding experimental data. Another study by Okaze et al. (2021) used the multi-model method. They studied the influence of using different SGS models on the simulation result of airflows around an isolated building. They compared the SSL, the DSL, the WALE, the coherent structure Smagorinsky (CSS) model, and a case using no SGS model. Their results agreed with the study by Liu, Niu, et al. (2019). Using or not using the different SGS models does not influence the mean velocities notably, but it does influence turbulent fluctuations. Moreover, in the study by Okaze et al. (2021), the simulated turbulence statistics are evaluated in terms of accuracy. Results indicate that using no SGS model achieves similar accuracy as using the SSL model, while the DSL, the WALE, and the CSS models improve the simulation accuracy to nearly equal extents.

Secondly, transient turbulent fluctuations need to be defined at the inflow boundary. Three different methods are commonly used in literature, namely the precursor method, the periodic method, and the synthetic method. Vortex method (VM) is a commonly used synthetic method, and it is readily available in CFD codes. In the study by Liu, Niu, et al. (2019), they reported that by increasing the number of vortices at the VM inflow boundary, the agreement between the simulation and the experiment improved, from *R* = 0.84 for 150 vortices to *R* = 0.90 for 230 vortices. Also, the distance between the inflow boundary and the building can introduce uncertainties to the simulation results. An upstream distance that is too small (equal to building height) deteriorates the simulation accuracy, but including a long upstream distance leads to ineffective use of computational resources (Liu, Niu, et al., 2019).

Thirdly, appropriate discretization schemes need to be specified. LES simulations differentiate between resolved and modeled scales using local grid scales. Since it is common to conduct mesh sensitivity tests in the investigated studies, uncertainties in this aspect can be minimized. Apart from the spatial discretization, Ikegaya et al. (2019) and Okaze et al. (2021) conducted sensitivity studies regarding influences of different numerical discretization schemes on simulation accuracy concerning airflows around an isolated building. Both of the studies reported that using the first-order upwind convection scheme notably deteriorated the overall simulation accuracy. In comparison, second-order schemes (central scheme in Ikegaya et al. (2019) and the linear scheme in Okaze et al. (2021)) are more accurate, but their accuracies deteriorated after blending with the first-order upwind scheme, especially for the accuracy regarding the turbulence statistics. The deteriorated accuracies result from the introduction of a high numerical viscosity in the first-order upwind scheme. Therefore, it is recommended to not use the first-order upwind scheme in conducting LES simulations for PLW studies.

##### Hybrid LES-RANS

**Fig. 5:**
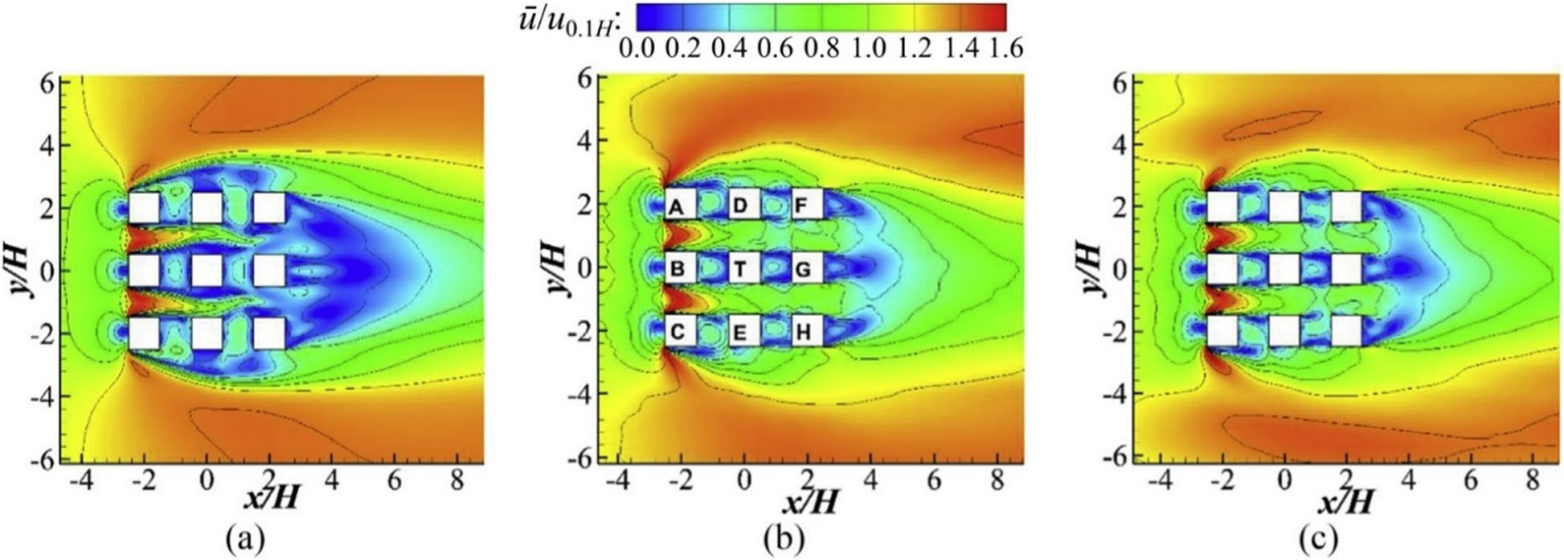
PLW flow fields simulated with a) SRANS, b) LES, and c) DES (cited from Liu and Niu (2019))

Predicting high Reynolds number flows using the LES approach can be a computationally prohibitive task. However, the computation cost can be reduced through modeling the near-wall region and resolving the outer layer only (Piomelli, 2008). Such approaches have been applied to investigate the high-fidelity transient PLW flow fields in different urban settings. Through multi-model comparisons, it is concluded that the hybrid LES-RANS approaches, e.g., detached eddy simulation (DES), offer significantly higher simulation accuracies compared to the SRANS (Liu et al., 2017; Vita et al., 2020) and the URANS approach (Liu et al., 2017) for obtaining the mean flow results. In comparison to the LES approach, their simulation results agree well (*R* = 0.90) for the simulated PLW flow field around a generic building array (Liu & Niu, 2019). Figure 5 shows the pedestrian-level mean flow fields around a generic building array (*Re* = 4.8 × 10^4^) simulated with SRANS, LES, and DES, respectively. It can be observed that DES has better agreement with LES than SRANS does. Further investigations identified the uncertainties embedded in hybrid LES-RANS simulations. Different turbulent fluctuations generating algorithms can lead to notable differences in the simulated PLW flow field around an isolated building with elevated design (a building design that has lift-up space at the ground level) (Liu et al., 2017). However, in a more complex urban setting, Vita et al. (2020) reported that the turbulent inflow profiles generated using different methods did not significantly affect the PLW flow field, indicating the local airflows are mainly affected by the complex geometrical features of the surrounding buildings.

Hybrid LES-RANS approaches are found to be more efficient compared to other simulation approaches. One typical example is delayed detached eddy simulation (DDES) (Spalart et al., 2006), which can make much better predictions than URANS using less calculation time (Liu et al., 2017). In modeling PLW flows around a generic building array, DDES is 1.2 times quicker than LES (Liu & Niu, 2019). Instead of using a blending function that depends on the flow field, the wall-modeled LES (WMLES) approach implements the RANS approach at the first wall-adjacent cell. Theoretically, coarse meshes used in RANS simulations are also allowed for running WMLES simulations. Despite the improved cost-effectiveness of the hybrid LES-RANS models, running hybrid LES-RANS simulations is still less practical for applications concerning more realistic large-scale urban areas. Vita et al. (2020) applied the WMLES approach to model the PLW flow field in the campus of the University of Birmingham (1.44 km^2^) within a time interval of 15 seconds. The simulation domain was discretized into 1.7 × 10^7^ cells spatially and 3 × 10^4^ time steps temporally. The calculation was conducted on a computer cluster using 140 central processing unit (CPU) cores and took 10 ~ 20 days of calculation time for different mesh resolutions. To sum up, the hybrid LES-RANS approach is an efficient and less computationally costly alternative to the LES approach. But, it is still an expensive technique that needs to be improved before it can be applied to large-scale realistic built environment cases.

##### Massively parallelized LES

We observe a growing trend of conducting LES simulations using massive parallelization codes. Most of the studies involved in this trend utilized mass. para. LES to investigate city-scale (above 1 km^2^) PLW flow fields at high-resolution (Fu et al., 2020; Wang et al., 2021; Zhang, Ye, et al., 2021). Typically, these studies used the non-commercial CFD code of the Parallelized Large-eddy Simulation Model (PALM) (Maronga et al., 2015) developed by the Leibniz University Hannover, Germany. The PALM model has been validated in several studies. For a generic urban area, good agreement with wind tunnel test results for mean velocities with buoyancy (*R*^2^ = 0.63) and without buoyancy (*R*^2^ = 0.67) is reported in Wang et al. (2021). In another study concerning roadside CO dispersion in a realistic urban area, the simulation accuracy varies with human activities and wind conditions (Zhang, Ye, et al., 2021). Their simulated CO concentrations agree well with the field measurements for non-rush hours and with middle or low natural winds (*R*^2^ = 0.35 ~ 0.67).

The feature that truly distinguishes the PALM model from other ordinary LES models is its linear speed-up effect with increasing number of CPU cores. The model is ready for running on graphics processing units (GPU). However, their actual simulations’ costs, i.e. computational resources as well as computing time are not clearly reported. In the study by Kristof and Papp (2018), who tested the Discovery Live software developed by Ansys, detailed simulation costs are partly provided. They modeled pollutant dispersion in a 3D street canyon for a 20 seconds time interval with the time step size of 5.2 × 10^− 4^ second and 9 million cells as computation domain. The simulation took approximately 1 h on a typical personal computer with a NVIDIA GTX 1080Ti graphics card. These mass. para. LES codes have great potential for PLW-related academic and practical built environment applications.

##### Lattice-Boltzmann method

**Fig. 6:**
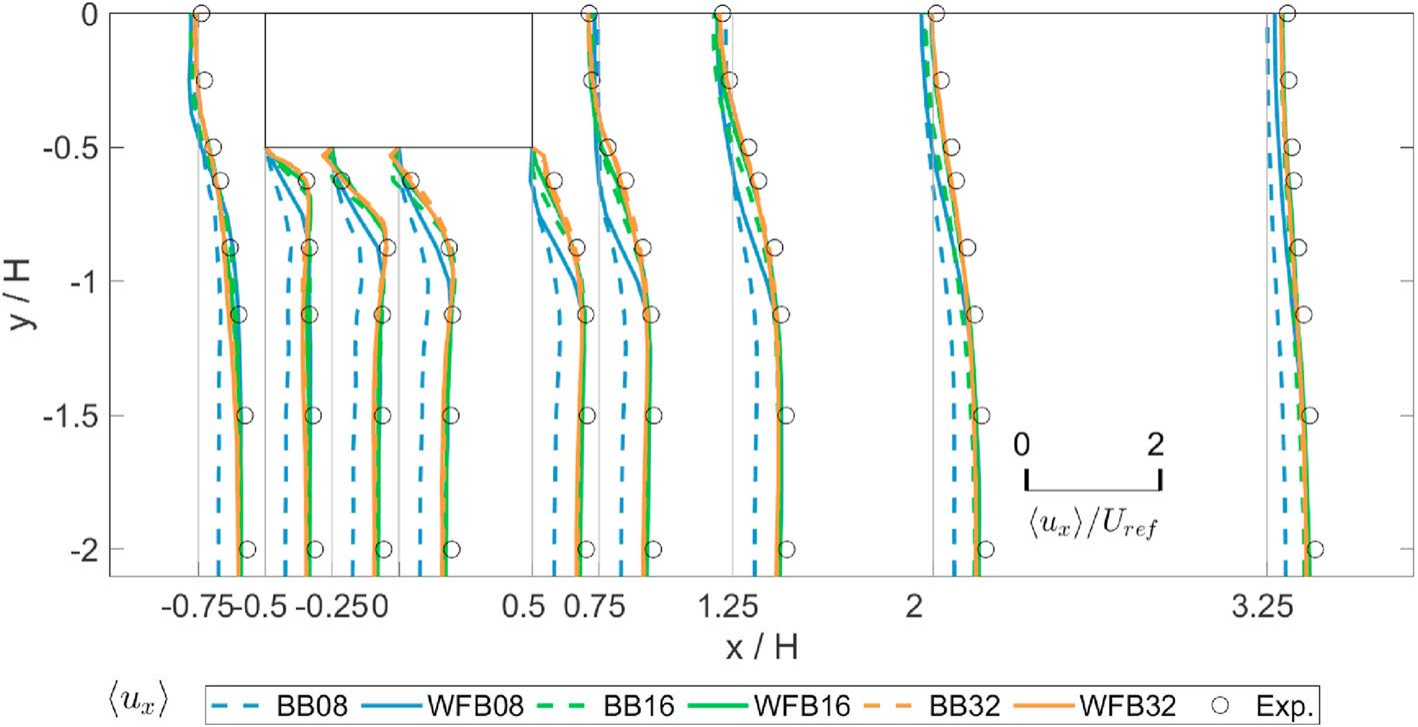
Mean streamwise velocity ratios around an isolated building simulated with LBM (cited from Han et al. (2021)). BB – bounce back boundary; WFB – wall-function bounce boundary; 08, 16, and 32 – coarse, medium, and fine mesh resolutions

The LBM approach simulates fluid flows by solving the lattice-Boltzmann equation (LBE), instead of the NSE (Chen & Doolen, 1998). Several studies have explored the application of LBM to simulate city-scale PLW flow fields. Potential influencing factors, including turbulence model, mesh resolution, time step size, and boundary condition, are found at the roots of uncertainties of the LBM approach. Similar to the LES approach, LBM resolves large-scale turbulent fluctuations and models small-scale turbulent fluctuations using SGS models. However, there is a lack in knowledge concerning how sensitive the LBM simulation results are to different SGS models. Uncertainties resulting from mesh resolutions can be minimized performing a mesh sensitivity test, which has become a necessary procedure for PLW simulation studies. As for the time step size, it appears that it can be determined in a deterministic way, based on the Courant–Friedrichs–Lewy (CFL) number and low Mach flow requirements (Ahmad et al., 2017; Merli et al., 2018). However, a sensitivity study of the time step is still advantageous allowing for possible improvements in cost-effectiveness. Last but not least, PLW flow fields are usually characterized by high Reynolds numbers. Other CFD models, e.g. URANS, can parameterize flow details near walls with wall functions to reduce computational costs. But for the LBM approach, there is commonly no appropriate wall function boundary condition implemented. Han et al. (2020) managed to incorporate the wall function boundary into LBM simulations by implementing a wall-function bounce boundary. The results indicate good agreement with experiments. Later, Han et al. (2021) conducted a sensitivity test on different wall boundary conditions for the LBM approach. As shown in Fig. 6, results showed a notably higher accuracy using the wall-function bounce boundary (blue solid lines) compared to the conventional bounce back boundary (blue dashed lines) when coarse meshes are used.

The LBM approach also has the advantage in running high-resolution simulations for PLW flow fields of large realistic urban areas with massive parallelization. For example, Mons et al. (2017) investigated the airflow in a compact urban area (1 × 1 km^2^) within a time interval of 3600 seconds, using 6 million cells and 10^5^ times steps. The calculation took approximately 8 hours for each run. Ahmad et al. (2017) modeled a coastal area of Tokyo (19.2 × 4.8 km^2^) using a high-resolution mesh that has approximately 1.2 × 10^10^ cells. A period of 4320 seconds was simulated on a GPU-based supercomputer using a time step size of 0.008 second, which took 40 hours of calculation time.

#### Fast fluid dynamics

**Fig. 7:**
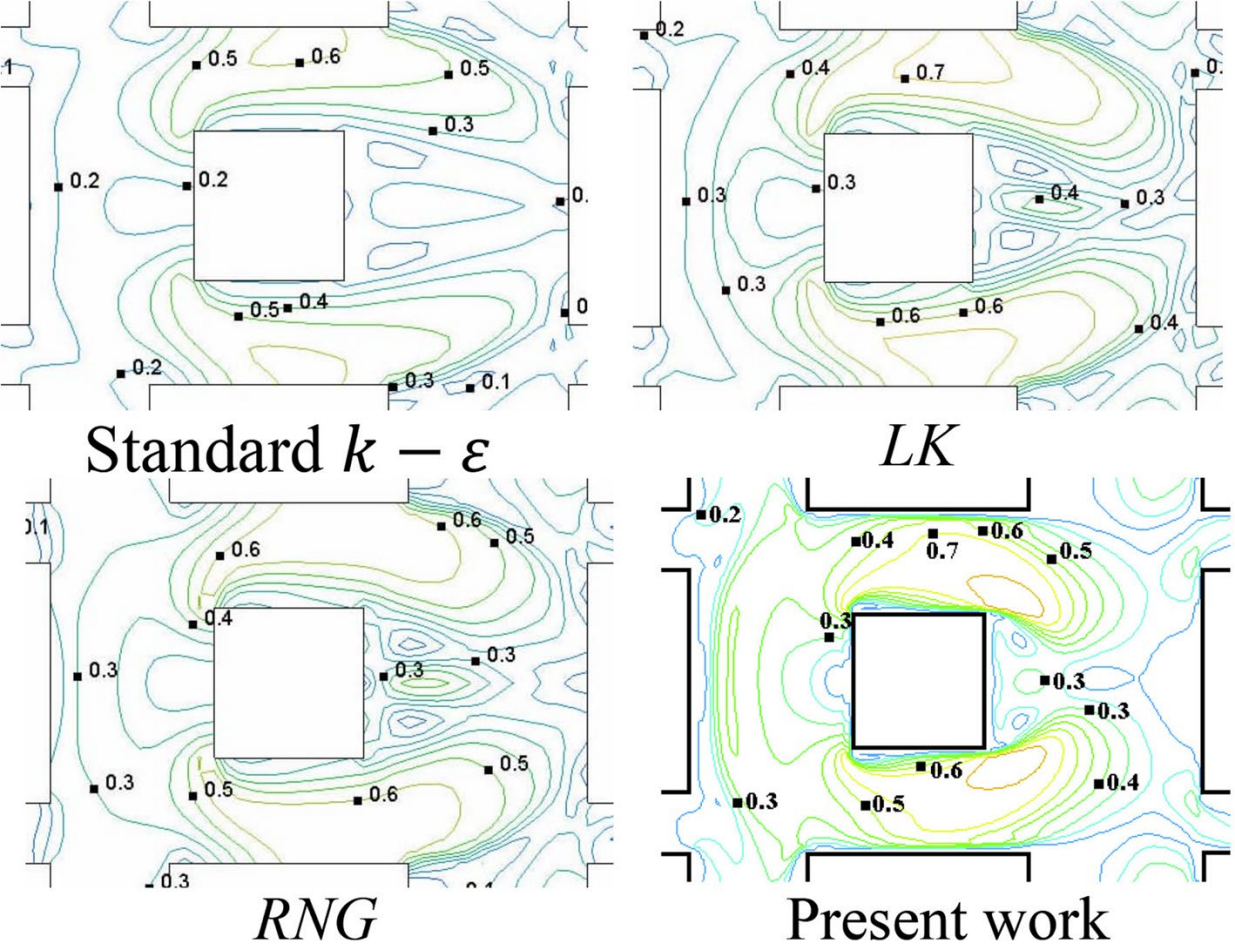
Normalized PLW mean velocities around the generic building array with a central high-rise building (cited from Mortezazadeh and Wang (2020))

FFD was first developed for fluid visualization in animation tools (Stam, 1999). In a previous study the FFD approach is applied to investigate the airflows around a building complex, but the simulation encountered convergence problems (Jin et al., 2013). Mortezazadeh and Wang (2020) recently modified a conventional FFD approach to implement large time steps and coarse meshes. Their simulation results agree well with wind tunnel tests of the PLW flow field around a building array with a central high-rise building. Comparisons are also made between the FFD model and three RANS simulations using the STD *k*-*ε*, the RNG *k*-*ε*, and the Launder-Kato (LK) *k*-*ε* turbulence models. As shown in Fig. 7, results indicate that the performance of the proposed FFD model is comparable to the RNG *k*-*ε* and the LK *k*-*ε* turbulence models for the prediction of normalized mean velocities at high wind regions, but overestimates the normalized velocities in wake regions. In comparison, the STD *k*-*ε* turbulence model underestimates the normalized velocities in the high wind regions, but making fairly good predictions regarding the normalized velocities in the wake regions.

It is worth noting that the FFD model, in the case of the study by Mortezazadeh and Wang (2020), took approximately 1.5 hours to simulate a validation case with 17 million cells, which could be considered significantly faster even than using the SRANS approach. Furthermore, their subsequent application case studied the transient PLW flow field in a complex urban area (9 km^2^), in which the unsteady simulation with 35 million cells took less than 2 hours of computing time. Given that FFD’s prediction accuracy has become an active research field recently (Dai et al., 2022; Li et al., 2022; Zheng et al., 2022), we may conclude that FFD shows potential for reducing computational time costs, but is still in further development and needs validation for more built environment cases.

### Data-driven models

In general, reduced-order modeling (ROM) can be used to enable near real-time flow simulations. Specifically, ROM is subdivided into intrusive ROM (IROM) and non-intrusive ROM (NIROM) models. IROM focuses on the projection of full-order governing equations onto low-order spaces in favor of higher solution efficiency (Fang et al., 2014; Galletti et al., 2004; Star et al., 2021; Tello et al., 2020). This approach, as the name indicates, is intrusive to the full-order governing equations as it involves manipulation of the governing equations. On the contrary, NIROM sidesteps the cumbersome procedures of manipulating and solving the governing equations. Specifically, NIROM uses either proper orthogonal decomposition (POD) or data-driven models to achieve the order reduction, and mostly uses data-driven models to retrieve full-order flow fields from the reduced-order representations. Given its close relation with the data-driven models, this review focuses on the NIROM approach. For more information about the IROM approach, readers are referred to the review by Masoumi-Verki et al. (2022).

**Fig. 8:**
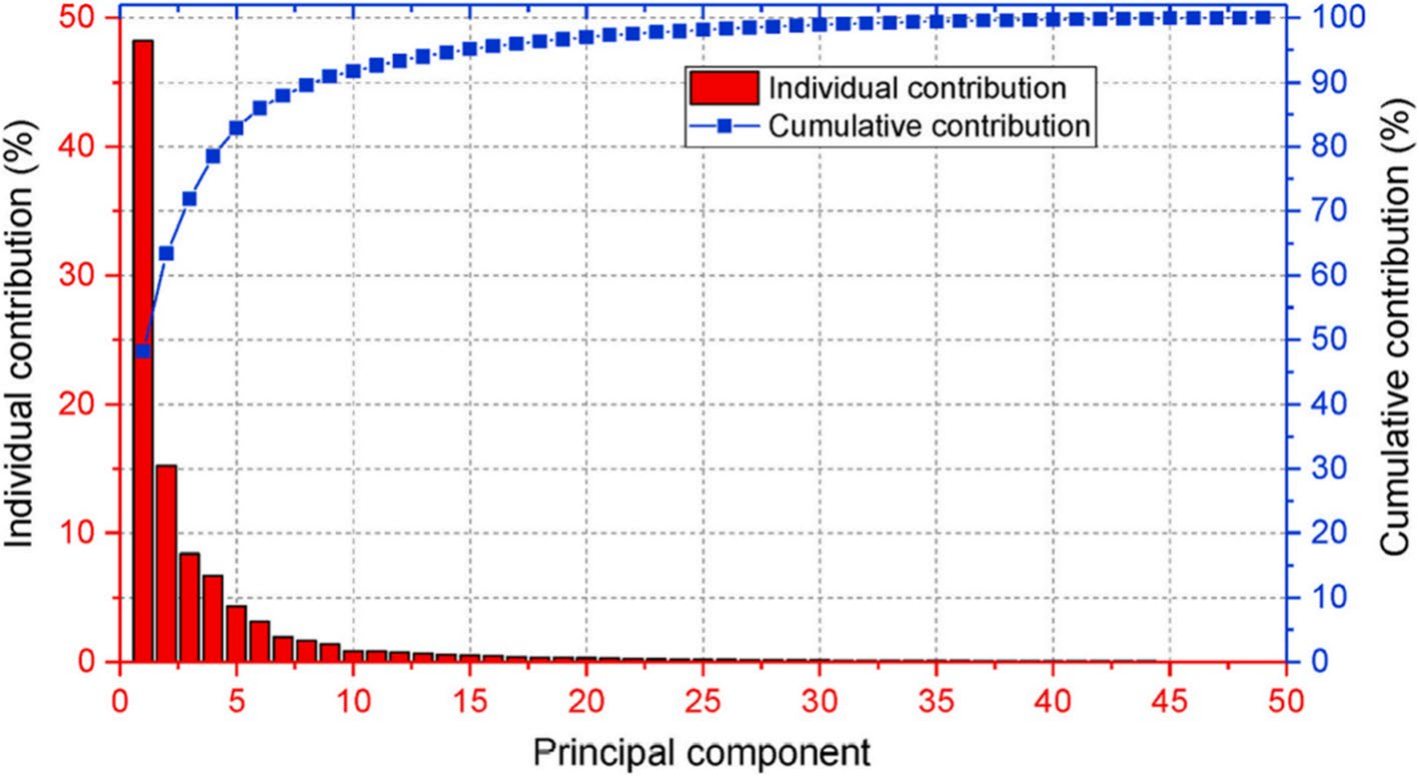
The first few principal POD modes kept in subsequent procedures for efficient computation and storage (cited from Weerasuriya et al. (2021))

To obtain the low-order representation of an unsteady PLW flow field using the POD method, the flow field **A** ∈ **ℝ**^N × S^ is decomposed into the POD mode matrix **U** ∈ **ℝ**^N × N^, the diagonal matrix **Σ** ∈ **ℝ**^N × S^, and the POD coefficient matrix **V**^T^ ∈ **ℝ**^S × S^. Their relations are typically expressed as follows: **A** = **UΣV**^T^. The superscript T refers to matrix transpose, N and S refer to the number of cells and the number of time steps contained in the full-order unsteady PLW flow field, respectively. Each column in **U** is a spatial mode for the full-order unsteady flow field, and the columns are arranged in a descending order with reference to the contribution to the full-order flow field. These spatial modes’ contributions are recorded in the diagonal elements of **Σ**. Only the first few modes that have adequate cumulative contributions are considered in the subsequent prediction procedures for better performance in terms of computation and storage. An example for the relation between the POD modes and the cumulative contribution is shown in Fig. 8, where the contribution of individual modes reduces, while the cumulative contribution increases. Information of the flow unsteadiness is stored in **V**^T^. Having obtained the low-order representation of the full-order flow field, future full-order flow fields can be predicted efficiently using data-driven models. The key is to train data-driven models that can predict future flow unsteadiness, i.e. predict new columns for **V**^T^. In this process, a variety of data-driven models can be used, and more discussion on this topic is available in Masoumi-Verki et al. (2022). In comparison with the high-fidelity simulation approach of LES, the NIROM approach can speed up the computing time by 10^5^ ~ 10^6^ times, as reported in the studies by Xiao, Heaney, Fang, et al. (2019) and Xiao, Heaney, Mottet, et al. (2019) in which unsteady flow fields in compact urban areas are studied.

**Fig. 9:**
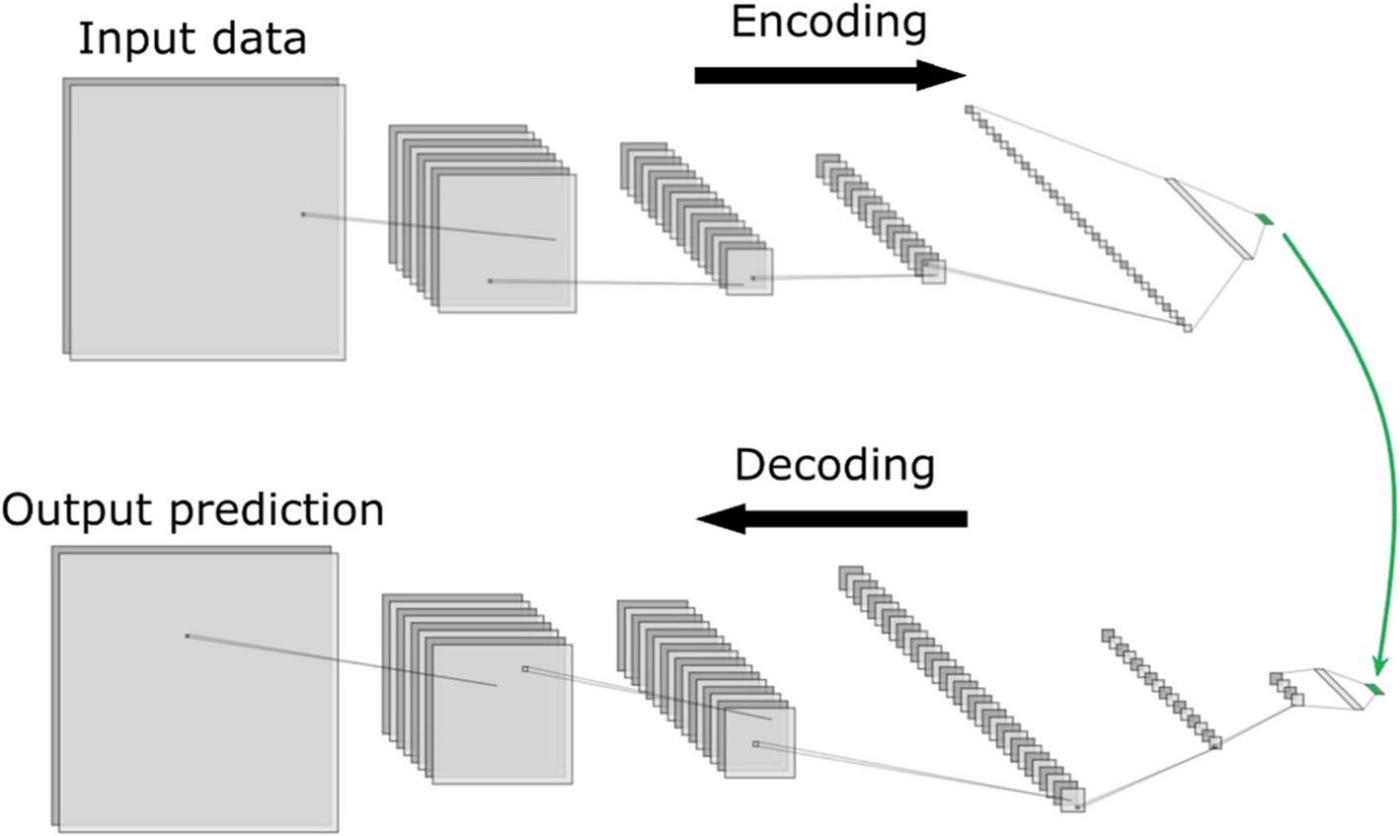
An example visualization of the workflow of using NIROM to predict full-order flow fields, where CNN is used for both regression and generation (modified from Xiang, Fu, et al. (2021))

Apart from using POD for order reduction, the low-order representations of full-order flow fields can also be obtained with data-driven methods. In this case, the low-order representations are found in the form of latent vectors in the so-called latent space. Specifically, a typical workflow for this type of NIROM method first involves the use of a data-driven model for regression on given boundary conditions to obtain the latent vector, and then another data-driven model known as the generator is used to retrieve the predicted full-order flow field from the latent vector. An example visualization of this workflow is shown in Fig. 9, where CNN is used for both regression and generation. The speed-up effect of this NIROM method is reported to be hundreds of times faster than the high-efficiency high-fidelity simulation approach of PALM, according to Xiang, Fu, et al. (2021) and Xiang, Zhou, et al. (2021) who focused on PLW flows in large urban areas.

Both NIROM methods show high efficiency and high accuracy. The major differences between these two methods are that POD-based NIROM has simpler expression, better interpretability, shorter training time, but fixed boundary conditions, while data-driven NIROM shows better accuracy, unsteady boundary conditions, but longer training time. It is worth noting that it is also possible to use POD on unsteady PLW flow fields with transient boundary conditions, but this will impair interpretability and lower prediction accuracy, as reported in the study by Xiang, Fu, et al. (2021) in which the high-fidelity unsteady PLW flow field around a large urban area was modeled with transient boundary conditions. NIROM models with four different order-reduction methods, namely CNN, MLP, linear regression, and POD, were compared and organized in descending order with respect to their prediction accuracies.

## Applicability of unsteady-state simulations in PLW comfort assessment

PLW comfort studies focus on the effect of winds on pedestrian wind comfort. Poorly designed PLW environments cause wind nuisance and affect pedestrians’ subjective feelings regarding the outdoor environment. In extreme cases, windy environment can cause pedestrians tripping over, exposing pedestrians to dangers (Blocken & Carmeliet, 2004). For a systematic PLW comfort assessment, three vital elements are required, namely wind statistics, wind amplification factors, and a wind comfort criterion (Blocken et al., 2016). Wind amplification factors reflect the impacts of surrounding built environment on local wind conditions, and are usually taken as the ratio of the local wind velocity to a reference wind velocity. The wind amplification factors are often obtained in an ad hoc manner. Field measurements provide discrete data points and wind tunnel measurements are expensive. Simulations can offer whole-flow field data at practical costs. Simulation approaches obtaining steady-state PLW flow fields, such as SRANS and some data-driven models, provide wind amplification factors for the translation of meteorology data to the building site. Broad applications of the steady-state simulations have proven that their tradeoffs between data fidelity and computation efficiency are acceptable (Dhunny et al., 2018; Ricci et al., 2022; Tsichritzis & Nikolopoulou, 2019). More discussions on PLW comfort assessment with SRANS can be found in the reviews by Blocken and Carmeliet (2004) and Blocken et al. (2016).

As observed in this review, there is recently a substantial growth of PLW studies using unsteady-state simulations. Their benefits to PLW comfort assessment are briefly discussed as follows. Using unsteady simulations, researchers can gain enriched insights into local wind conditions. For example, gust wind velocities that are commonly used in PLW comfort assessment can be derived from the unsteady simulation results accurately (Jacob & Sagaut, 2018). However, running unsteady-state simulations would incur extra computational costs, especially for the high-fidelity models. LES is a highly accurate simulation approach, but it often induces impractical computation costs. Hybrid LES-RANS approaches, such as DES and WMLES, are versions of the LES approach that have incorporated tradeoffs for higher computation efficiency, but they are still far from being a practical option for practical city-scale built environment applications, especially for those involving multiple alternative design options. On the other side of the computational speed - accuracy spectrum, there is the FFD approach which is computationally efficient but still introduces too many uncertainties for the wind comfort assessments due to the mediocre simulation accuracy. In particular, there are studies promoting large time steps to further increase FFD’s computational efficiency. But, these choices lower the time resolution undermining the approach’s applicability.

Other discussed simulation approaches appear more promising for PLW simulations in large realistic urban areas. Allegedly, the mass. para. LES approach could achieve linear speed-up with increasing CPU cores. Also rapid LES simulations are to be considered if there is enough CPU cores within one’s access. However, for the mass. para. LES approach, the studies investigated in the present review do not provide sufficient information about their actual computational costs, with exception for the study by Xiang, Zhou, et al. (2021). They documented that the computation time for a city-scale PALM simulation within a one-day time interval would be approximately 22 days running on Intel(R) Xeon(R) Platinum 8160 CPU processors with 48 cores. Therefore, mass. para. LES can be considered as a possible practical option for practical built environment applications concerning PLW comfort if shorter time intervals are considered and enough CPU cores are provided.

LBM can also be considered as a promising approach. Ahmad et al. (2017) applied the LBM approach to simulate unsteady PLW flow field in a city (19.2 × 4.8 km^2^) at both high spatial (1.2 × 10^10^ cells) and high temporal (0.008 s) resolution. Running on a GPU-based supercomputer, the simulation of the time interval of 1.2 h took 40 h of computation time. Because of the different computer specifications and the insufficient information on numerical settings, the computation efficiencies of mass. para. LES and LBM are not compared.

Substantial speed-ups achieved by data-driven models are observed in this review. With the high computation efficiency, it is safe to say that the data-driven model can predict a city-scale PLW flow field in real-time. However, at the current state the major restriction for applying the data-driven model in practical planning and design applications is the precursor time needed for obtaining the training data and training the model. In the study by Xiang, Zhou, et al. (2021), it took 4 weeks to obtain the training data set for a given urban setting using a high-efficiency PALM model. For applications concerning generic building geometries, there might be available pre-trained models (Weerasuriya et al., 2021), and these models can be recommended to be more computationally effective.

**Fig. 10:**
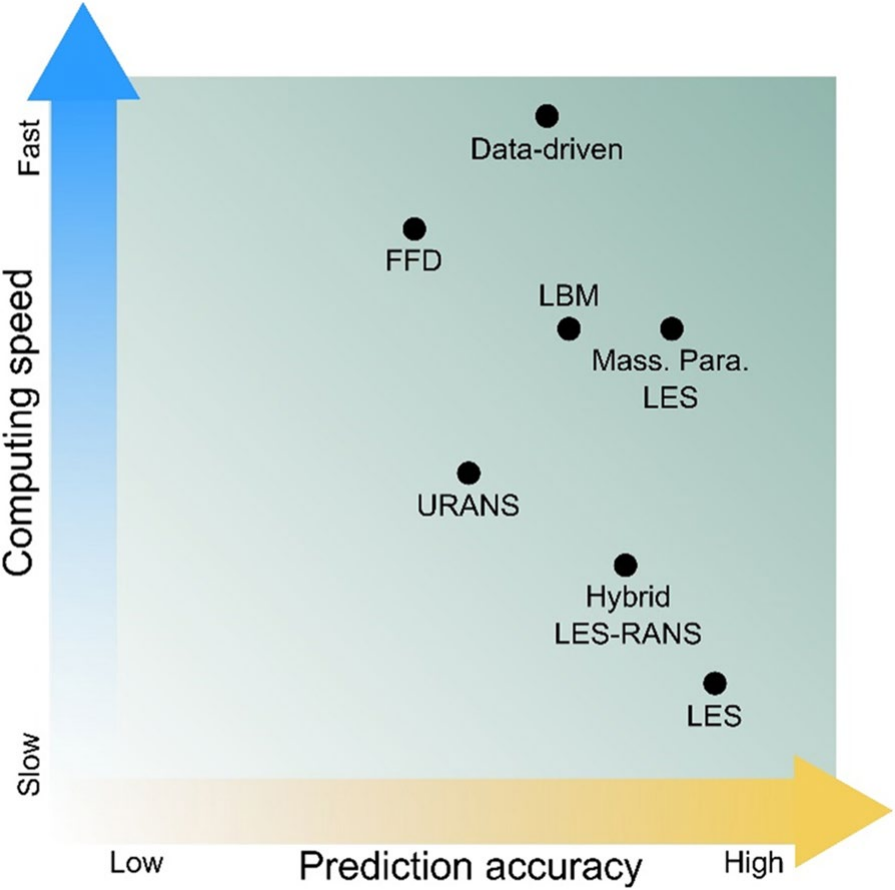
Qualitative presentation of prediction accuracy versus computing speed of reviewed simulation approaches for unsteady-state PLW studies

Finally, we summarize the aforementioned discussions by marking the discussed unsteady-state simulation approaches on a prediction accuracy - computing speed diagram, as shown in Fig. 10. The abscissa refers to the prediction accuracy, and the ordinate refers to the computing speed. It is worth noting that the data-driven model appears at the top of the plot because it is presumed to be trained, or in the online prediction stage, after training with high-fidelity data. And, it is also presumed that there are adequate computational resources, i.e. CPU or GPU cores and storage, for LBM and mass. para. LES, so they appear in the top half on the plot.

## Conclusions

We reviewed the use of CFD and data-driven models for pedestrian-level wind (PLW) simulation studies as reported in the literature in last 5 years. Emerging trends, advances, and challenges of different simulation approaches in this field are discussed and articulated critically with a focus on the computational efficiency and accuracy. The main conclusions are as follows:

1. SRANS is still the most dominant simulation approach. Among the 215 CFD studies investigated in this review, 62.3% of them used the SRANS approach. However, SRANS simulations have to be used with caution because of certain uncertainties embedded in the approach. It is recommended, as has been done successfully in the studies investigated in this review, to minimize the uncertainties by conducting sensitivity tests for model closure coefficients or performing multi-model comparative studies for choosing the most appropriate turbulence models for the current application.
2. There is a thriving trend of conducting unsteady-state simulations with high-efficiency approaches. Apart from the conventional URANS and LES approaches, hybrid LES-RANS, mass. para. LES and LBM have been preliminarily assessed and applied in modeling turbulent wind flow in the built environment. They show improved computational efficiencies and promising simulation accuracies.
3. The pre-trained data-driven model has unmatched computational efficiency in predicting PLW flow fields after a successful training of the model using high-fidelity simulation results. However, at the current stage, when access to pre-trained data-driven models is still limited, the precursor time required for obtaining the high-fidelity training data for the specific application and training the model still render the data-driven model impractical for urban planning and design applications. Nevertheless, the pre-trained data-driven models show an important potential in future to perform fast and accurate simulations for the wind environment at the pedestrian level.
